# Multiplicity of α-Synuclein Aggregated Species and Their Possible Roles in Disease

**DOI:** 10.3390/ijms21218043

**Published:** 2020-10-28

**Authors:** Pablo Gracia, José D. Camino, Laura Volpicelli-Daley, Nunilo Cremades

**Affiliations:** 1 Joint Unit BIFI-IQFR (CSIC), Institute for Biocomputation and Physics of Complex Systems (BIFI), University of Zaragoza, 50018 Zaragoza, Spain; pgracia@bifi.es (P.G.); jcamino@bifi.es (J.D.C.); 2 Center for Neurodegeneration and Experimental Therapeutics, Department of Neurology, University of Alabama at Birmingham, Birmingham, AL 35294, USA; lvolpicellidaley@uabmc.edu

**Keywords:** α-synuclein, amyloid aggregation, oligomer, fibril, polymorph, neurodegenerative disorders, synucleinopathies

## Abstract

α-Synuclein amyloid aggregation is a defining molecular feature of Parkinson’s disease, Lewy body dementia, and multiple system atrophy, but can also be found in other neurodegenerative disorders such as Alzheimer’s disease. The process of α-synuclein aggregation can be initiated through alternative nucleation mechanisms and dominated by different secondary processes giving rise to multiple amyloid polymorphs and intermediate species. Some aggregated species have more inherent abilities to induce cellular stress and toxicity, while others seem to be more potent in propagating neurodegeneration. The preference for particular types of polymorphs depends on the solution conditions and the cellular microenvironment that the protein encounters, which is likely related to the distinct cellular locations of α-synuclein inclusions in different synucleinopathies, and the existence of disease-specific amyloid polymorphs. In this review, we discuss our current understanding on the nature and structure of the various types of α-synuclein aggregated species and their possible roles in pathology. Precisely defining these distinct α-synuclein species will contribute to understanding the molecular origins of these disorders, developing accurate diagnoses, and designing effective therapeutic interventions for these highly debilitating neurodegenerative diseases.

## 1. Introduction

**Figure 1:**
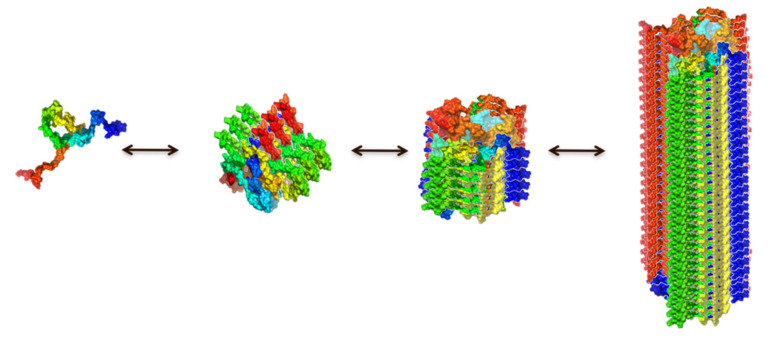
Schematic representation of the process of amyloid formation according to a nucleation-conversion-polymerization model. This model has been proposed for the process of αS aggregation when triggered at conditions of heterogeneous primary nucleation [10]: the initially formed oligomers slowly convert into partially formed β-sheet oligomers that further elongate and generate fully-formed mature fibrils. Note that this is a very simplified linear representation of the real funnel-like conformational landscape of the process.

Many neurodegenerative disorders, including Alzheimer’s disease, Parkinson’s disease and prion disease, are characterized by protein inclusions that are formed by the conformational conversion of normally soluble proteins or peptides into oligomeric intermediates and eventually amyloid aggregates and fibrils by a process referred to as amyloid aggregation [1,2] (Figure 1). Amyloid aggregates, typically with a fibrillar morphology, are protein self-assembled structures composed primarily of one type of protein or peptide, which adopt a characteristic structural architecture, termed the cross-β structure [3,4,5,6,7,8]. This particular structure consists of arrays of extended β-sheets that run the length of the fiber, in which individual β-strands are arranged in an orientation perpendicular to the fibril axis [3,4,7]. The molecular mechanisms by which proteins adopt this structure is of unquestionable interest, and much progress has been recently made through the development of new experimental approaches, and by combining experimental and theoretical methods using the formalism of chemical kinetics [9,10,11,12]. However, there are still important questions that remain to be clarified such as how and why a specific protein starts to self-assemble, how the acquisition of the amyloid structure occurs, and how this process induces toxicity.

The pathogenicity of amyloid formation has been associated with both a loss of function of the proteins that aggregate, and a gain of toxic function through the generation and accumulation of aggregated forms of the protein [2,13], which spread within cells and propagate toxicity from cell to cell [14,15]. Preventing the progression and the toxicity associated with amyloid formation, however, requires precise definitions of the protein species that are toxic, and those that are able to spread and recruit more protein units into toxic amyloid aggregates. There is continuous discussion as to which protein aggregated species are more damaging to cells, either the fibrillar-end products of the aggregation reaction or the soluble oligomeric intermediate species [10,13,16,17,18,19,20,21]. Both oligomeric and fibrillar species of multiple proteins and peptides can induce toxicity by similar mechanisms including membrane perturbation, calcium and metal ion imbalance, oxidative stress, and overload of chaperone and ubiquitin proteasome systems [22,23,24,25,26,27,28,29], which suggests generic aggregation and toxicity pathways between different amyloidogenic proteins and peptides [1,13], as well as possible common mechanisms of toxicity between oligomeric and fibrillar species.

The understanding of the role of the various aggregated species on neurodegeneration has increased even more in complexity with recent studies showing that oligomeric species can be generated via fibril disaggregation processes [10,30,31] and by secondary nucleation mechanisms where the fibrillar surface can catalyze the formation of oligomeric species [32,33]. These results suggest that the fibrillar species and amyloid inclusions can act as a source of soluble oligomeric species, which in turn can generate new fibrillar species. At the same time, variations in the solution conditions or the particular cellular context that the protein encounters leads to structurally different amyloid conformations, also referred to as polymorphs, which have been associated with specific types of pathologies [18,34,35].

In this review, we will focus on the current knowledge of the multitude of α-synuclein (αS) aggregated species generated through amyloid self-assembled processes and the possible roles these species could have in the development and spreading of neurodegeneration, and in the induction of distinctive types of synucleinopathies.

## 2. α-Synuclein Aggregation and Synucleinopathies

αS is an intrinsically disordered protein of 140 amino acids, widely expressed throughout the body, particularly in the central nervous system, including the dopaminergic neurons of the substantia nigra pars compacta (SNc), excitatory neurons in the cortex, amygdala and olfactory bulb and inhibitory neurons in the globus pallidus, subthalamic nucleus and substantia nigra pars reticulata [36]. Its primary sequence can be divided into three regions. The N-terminal domain (residues 1–60), which has a predisposition to fold into amphipathic α-helices, particularly upon interaction with lipid membranes [37,38]. The central hydrophobic region (residues 61–95), named nonamyloid β component (NAC) region (due to historical reasons to differentiate this amyloid-prone region in αS from the amyloid β peptide involved in Alzheimer′s disease [39,40]), which has a predisposition to fold into either α-helix conformation upon interaction with highly negatively charged lipid membranes [41,42], or β-sheet structure upon self-assembly [43]. And the proline-rich and highly negatively charged (at neutral pH) C-terminal region (residues 96–140), with no structure-forming propensity. There has been some controversy as to what would be the native conformation of the protein at physiological conditions [44]. The most accepted current paradigm is that the protein remains unfolded (as an intrinsically disordered protein, IDP) [45,46] in the cytosol, although with some tertiary contacts between the C-terminal and the NAC and N-terminal regions of the protein [47,48]. Approximately 1/3 fraction of αS in neurons adopts a partially folded α-helical structure [49] upon binding to membranes [50]. The functional conformation of the protein, at least for its role in synaptic vesicle trafficking and neurotransmission release, has been proposed to consist on α-helix-rich oligomers that are assembled on the cellular membranes [49]. However, under pathological conditions, αS self-assembles into amyloid aggregates, with the typical cross-β structure, which can ultimately form amyloid-rich inclusions.

The presence of these amyloid inclusions is the histopathological signature of a number of neurodegenerative disorders collectively referred to as synucleinopathies [51,52]. The three major synucleinopathies include Parkinson’s disease (PD), dementia with Lewy bodies (DLB) and multiple system atrophy (MSA). PD is characterized by motor symptoms such as tremor at rest, slowness of movement, and balance problems. Up to 80% of PD patients develop cognitive changes called PD-dementia [53,54]. Patients with DLB initially present cognitive changes and hallucinations prior to the development of parkinsonism. MSA is characterized by similar motor defects to PD as well as cerebellar ataxia, and autonomic failure. MSA is more similar to prion diseases in that it rapidly progresses and life expectancy is much shorter compared to PD. In addition, a significant proportion of Alzheimer´s disease patients also have αS pathology, although in most cases restricted to the amygdala, unlike synucleinopathies [55].

PD is the second most common form of neurodegeneration following Alzheimer´s disease. The mean age of onset of the sporadic forms of the disease, which represent 90% of the cases, is approximately 65 years old. In the hereditary forms associated with the αS gene (SNCA), however, there is a much earlier onset of the disease (the most potent mutation being the G51D) with a faster and more severe progression of pathology [56,57,58]. αS gene (SNCA) duplication, triplication and polymorphisms also cause PD [59,60,61]. Autosomal dominant inherited mutations in genes including LRRK2 and GBA cause late onset PD with symptoms similar to sporadic PD, while autosomal recessive mutations in other genes such as PINK1 cause early onset forms of parkinsonism [62,63]. LRRK2 and GBA pathological mutations have been reported to increase the formation of αS inclusions [64,65,66,67]. It is important to note that not all individuals with LRRK2 mutations show neuronal αS inclusions in postmortem brains, and whether individuals with autosomal recessive mutations have αS pathology (typically referred to as Lewy body pathology) remains questionable. The typical motor symptoms of the disease are a consequence of the degeneration of dopaminergic neurons in the SNc in the basal ganglia of the brain resulting in bradykinesia and rigidity [68]. In addition, there are neuropsychiatric symptoms that occur at later stages, and other non-motor symptoms that have been recently suggested to occur at the early stages of the disease [68].

αS inclusions inside neurons are referred to as Lewy bodies (LBs) and Lewy neurites (LNs) in PD and DLB. In MSA, however, αS accumulates primarily into inclusions found in the cytosol of oligodendrocytes, called glial cytoplasmatic inclusions, although a small percentage of neurons can also have αS inclusions [51]. The aggregation of αS into amyloid aggregates is thought to play a key role in the initiation and spreading of these diseases, although controversy remains whether LBs or smaller polymorphs of aggregates contribute to neuronal defects and toxicity. αS is not only pathologically but also genetically linked to disease. Hereditary mutations in the SNCA gene or duplications and triplications of the wild-type gene lead to autosomal dominant forms of PD and DLB, with an earlier age of disease onset [59,69,70]. In these familial cases, accumulation of αS in LBs and LNs is also observed [51,71], and the cases of triplications in the SNCA gene typically result in a more severe disease progression than in the cases of gene duplication. In addition, a genome-wide association study has shown that individuals with certain polymorphisms of the SNCA gene have a higher risk of developing sporadic PD and MSA [60,72], and some of these polymorphisms have been linked to a higher expression of αS in neurons [73]. Further, the injection of αS amyloid fibrils into the brains of healthy mice induce αS inclusion formation and PD-like pathology [14,74,75].

The genetic evidence, along with the neuropathologic evidence for accumulation of αS in patients with PD and other synucleinopathies, indicates a central role for αS in the pathogenesis of both the inherited and sporadic forms of these diseases. Indeed, evidence suggests a mechanistic link between even slightly higher levels of αS and the formation of αS amyloid aggregates within neurons and the induction of neurodegeneration, likely through the generation and accumulation of toxic αS aggregated species during the process of amyloid self-assembly [76,77].

## 3. α-Synuclein Aggregation Mechanisms and Pathways

**Figure 2:**
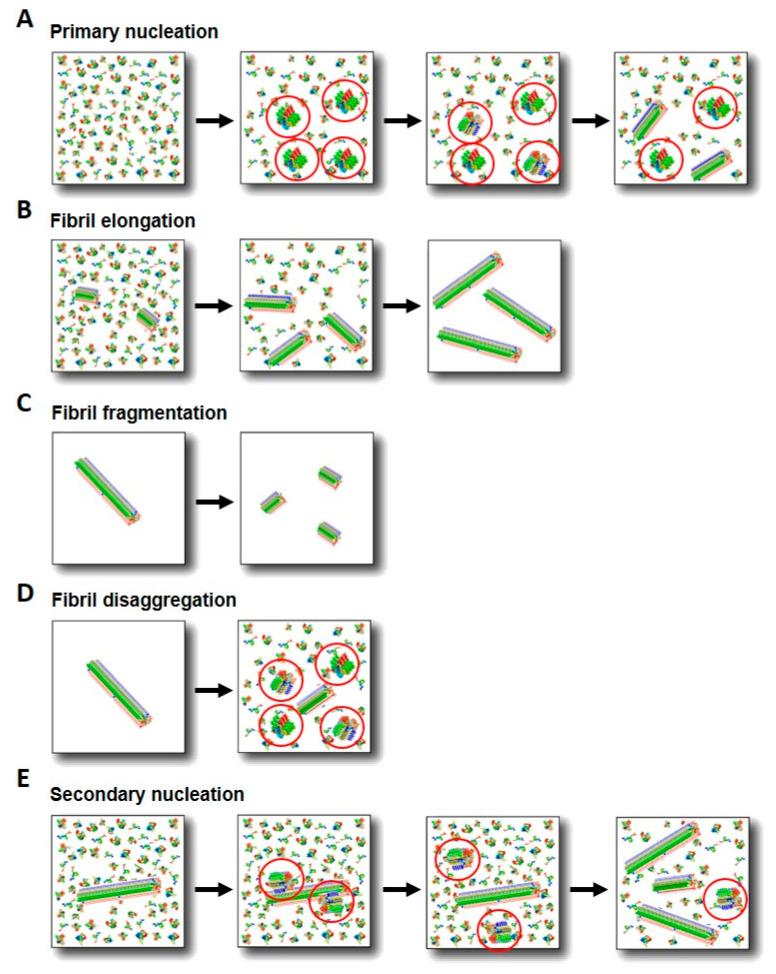
Schematic representation of the different processes that can take place during amyloid fibril formation. Oligomeric species, highlighted with circles for a better visualization, can be generated through primary nucleation (**A**), but also through fibril disaggregation (**D**) or secondary nucleation (**E**) processes. Fibril elongation (**B**) and fragmentation (**C**) are also represented.

The transition of a protein from its functional, typically monomeric state to the amyloid state is a highly complex process that depends on both intrinsic features of the particular protein and the environmental conditions. Early analysis of in vitro kinetics of formation of amyloid fibrils show that the overall process includes a nucleation step (Figure 1 and Figure 2A), where oligomeric species and a sufficient number of fibril nuclei are formed, followed by an exponential step, reflecting the nuclei-dependent growth through monomer addition, generating protofilaments and eventually mature amyloid fibrils. This type of mechanism has been described by a nucleation-polymerization model [78]. The typical sigmoidal kinetic profiles observed for amyloid formation were interpreted as a greater ease of monomer addition onto already formed aggregates compared to the *de novo* formation of aggregates directly from monomers through nucleation. Consequently, by adding preformed fibrils to a monomeric solution, the nucleation step is bypassed, and the kinetic curves show only the exponential growth step, typically reflecting the elongation process (Figure 2B). The growth of the fibrils by monomer addition onto the fibrillar ends ensures that the structural properties adopted by the newly added protein molecules are identical to those of the molecules of the fibrillar parents in a phenomenon called templating [79], which is analogous to that observed in crystallization. More complex models have been later developed for analyzing the amyloid aggregation mechanism of certain amyloidogenic proteins. One such model is the so-termed nucleation-conversion-polymerization model which includes a structural conversion of the early formed oligomers into β sheet-enriched, elongation competent oligomers [80,81] (see Figure 1).

### 3.1. Primary Nucleation

Aggregation of αS is typically initiated in vitro by subjecting the protein solution to agitation, either shaking or stirring [82]. Under diluted, highly hydrating conditions, at neutral pH and physiological ionic strength and temperature, αS is not observed to aggregate for more than 7–10 days of incubation without sample agitation. However, when the sample is strongly agitated, aggregation is triggered within 1–3 days, depending on protein concentration and agitation speed. In those conditions, nucleation initiates at the air/water interface or any other hydrophobic/hydrophilic interface present in the sample, such as the hydrophobic coatings of sample containers or stirring bars, by heterogeneous primary nucleation [83,84]. The role of agitation in promoting aggregation could be altering and/or increasing the surface of the nucleation-active air/water interface, and increasing the apparent elongation rate through the multiplication of growth-competent aggregate sites by shearing-induced fibril fragmentation. Given the propensity of the N-terminal region of αS to acquire amphipathic α-helices, the protein preferentially partitions or adsorbs at hydrophobic/hydrophilic interfaces, in order to simultaneously maximize the hydrophilic interactions in the aqueous environment and the hydrophobic force at the hydrophobic surface [85]. At the interface, the protein initiates self-assembly (under highly hydration conditions), likely as a result of the local increase in protein concentration and the selection of nucleation-efficient conformations upon adsorption. This feature has been indeed used to develop other strategies of inducing aggregation, such as the addition of hydrophobic nanoparticles [86], or larger beads [87], or even lipid vesicles composed of particular (typically non-physiological) types of phospholipids, which have resulted in good nucleation-active surfaces [88,89]. Interestingly, this is not a unique property of αS, since hydrophobic/hydrophilic interfaces have been found to be critical for the aggregation of many other amyloidogenic proteins and peptides, including IDPs such as the Aβ peptide [90], and folded proteins such as insulin [91].

We have recently observed, however, that αS can form amyloid aggregates without the need of a nucleation-active surface through homogeneous nucleation under limited hydration conditions, and that when the protein undergoes this process, there is a preference for remarkably different amyloid polymorphs. Specifically, there is a preference for an antiparallel β-sheet arrangement, in contrast to the parallel β-sheet architecture adopted when heterogeneous nucleation dominates [92]. The formation of amyloid aggregates rich in intermolecular antiparallel β-sheets under limited hydration conditions has been also reported for other amyloidogenic peptides, and a multitude of a priori non-amyloidogenic proteins belonging to different structural classes, as well as disordered peptides such as poly(L-lysine) [83,93,94,95,96,97,98,99].

The antiparallel intermolecular β-sheet structure has been also previously observed in stable, particularly toxic oligomers of αS and other amyloidogenic systems and has been proposed to be distinctive of these toxic species [100,101,102,103]. These oligomers have been suggested to be off-pathway by some researchers. In the light of our new findings, they are, however, best described as on-pathway of an amyloid aggregation process triggered by homogeneous nucleation under limited hydration conditions. We noticed that a significant number of protocols to generate the stable antiparallel β-sheet oligomers reported include a lyophilization step or the peptide/protein stock is lyophilized. These stable oligomers represent a good model to investigate the structural and biological properties of pre-fibrillar amyloid oligomers, bearing in mind that some of their properties might differ significantly from those of the parallel β-sheet oligomeric intermediates generated under the typical in vitro aggregation reactions (i.e., at the air/water interface), which are more difficult to trap.

One of the cellular microenvironments with a particularly low content of water (by definition) is the interior of protein-rich droplets, generated by liquid-liquid phase separation (LLPS). These phase separated protein droplets likely play a role in the in vivo aggregation of a number of amyloidogenic proteins such as tau [104,105], TDP-43 [106,107] and αS [108,109]. Although the mechanism of the liquid-to-solid transition of these protein droplets is unclear, our recent data on the amyloid aggregation inside αS droplets generated in vitro suggest that the process is triggered by homogeneous primary nucleation resulting in the formation of amyloid aggregates with a preference for an intermolecular antiparallel β-sheet structure [92]. Whether this mechanism occurs in the liquid-to-solid transition of αS droplets in vivo and can take place in other protein-rich droplets of other amyloidogenic proteins needs to be explored.

### 3.2. Secondary Processes

In addition to primary nucleation (Figure 2A) and templating-based aggregate elongation (Figure 2B), other process have been shown to be important in the overall amyloid aggregation process, insofar they give raise to different αS self-assembled species, including fibril fragmentation, disaggregation and fibril-catalyzed secondary nucleation [10,12,110,111,112]. Fibril fragmentation (Figure 2C) implies the rupture of elongated fibrils into smaller fragments, thus resulting in the multiplication of growth-competent fibrillar ends accessible for monomer addition. This can occur at any phase of the self-assembly process [113] and significantly accelerates the apparent fibril growth rate [12]. Prolonged exposure of fibrils to mechanical stress results in the decrease of their length distribution toward a limit that is solely determined by the mechanical properties of the fibrils [114], while maintaining their fibrillar nature. There is no evidence, therefore, that fibril fragmentation can originate significant fractions of oligomeric species. This process is greatly enhanced by shear forces such as those existing in typical αS in vitro aggregation setups, but its effect in vivo remains unclear.

Oligomeric species are typically assumed to be formed during primary nucleation (Figure 2A), but they can also be generated through secondary processes. The release of αS oligomeric species from fibril disaggregation (Figure 2D) has been observed in vitro upon dilution of fibrils [10,112,115], with significant fractions of oligomeric species generated when the fibrils are at concentrations below approximately 1 µM [10]. This process that has been observed in vitro, could be favored in the cellular context through the disaggregation activity of chaperones (see Section 6) or other fibril destabilizing agents [30] and could be highly relevant in the cell-to-cell transfer if fibrils are released to the extracellular space.

αS amyloid fibrils can be formed with a greatly reduced lag-phase duration by secondary nucleation processes catalyzed at the surface of the amyloid fibrils (Figure 2E). This process implies the *de novo* assembly of monomers onto the surface of pre-assembled aggregates and represents, therefore, a particular type of heterogeneous nucleation. The aggregation of the Aβ42 peptide into amyloid fibrils under typical physiological conditions in vitro has been shown to be dominated by secondary nucleation processes, once primary nucleation has been triggered [116]. The consequence is a significant amplification of the number of aggregates during the reaction, with the generation of significant concentrations of toxic oligomers [32]. Therefore, secondary nucleation processes have been suggested to play an important role in amyloid spreading in disease scenarios [110,117,118]. In the case of αS, secondary nucleation occurs at mildly acidic pH conditions (under highly hydration conditions) [118], such as those present in endosomes and lysosomes in the cell, and its relevance in vivo remains unknown.

### 3.3. Physicochemical Factors Affecting Amyloid Aggregation Process

A number of physicochemical factors in the solution conditions have been proposed to modulate not only the rate but also the route of amyloid formation in αS and other amyloidogenic proteins and peptides. Different amyloid polymorphs are accumulated upon slight variations in the solution conditions of the aggregation reaction. Among all the factors, three appear to be key in αS: pH, ionic strength and water activity. pH has been shown to modulate the relative rates of elongation and secondary nucleation processes, which not only can affect the structure of the most populated amyloid polymorphs, as explained below, but also the overall rate of aggregate multiplication and, therefore, in vivo spreading [118]. The differences in ionic strength seem to affect primarily the folding and packing of the fibrillar structure and thus the preference for a particular amyloid polymorph [119]. In addition, relatively high ionic strengths, including physiological concentrations, increase the formation of higher-order assemblies of fibrils [118], an effect that might be relevant during LB formation. Water activity and, therefore, the protein hydration state have been recently demonstrated to be a key determinant not only for triggering αS self-assembly (maintaining αS monomeric and preventing it from misfolding and self-assembly under highly hydration conditions), but also for dictating the preference for the type of primary nucleation (heterogeneous vs homogeneous) and the type of structural amyloid polymorph generated (parallel vs antiparallel β-sheet structure). Conditions of very poor water activity such as those encountered inside protein-rich droplets, generated by LLPS, have been reported to be particularly efficient in triggering αS amyloid aggregation both in vitro and in vivo [105,108,109]. 

Depending on the microenvironment that αS encounters in the cell, therefore, alternative amyloid aggregation mechanisms and pathways would be triggered, leading to the formation of remarkably different amyloid polymorphs.

## 4. Multiplicity of α-Synuclein Aggregated Species

Two distinct major pools of protein aggregated species can be distinguished during the general process of amyloid aggregation: soluble oligomers, and insoluble fibrillar species. Each of these pools, however, encompasses an array of individual species both in terms of size and structure. In addition, significant variability in terms of ultrastructure has been observed between amyloid aggregates, both in terms of oligomers and fibrils, even if they have similar sizes and secondary structure contents [119,120].

### 4.1. Fibrillar Polymorphs

**Figure 3:**
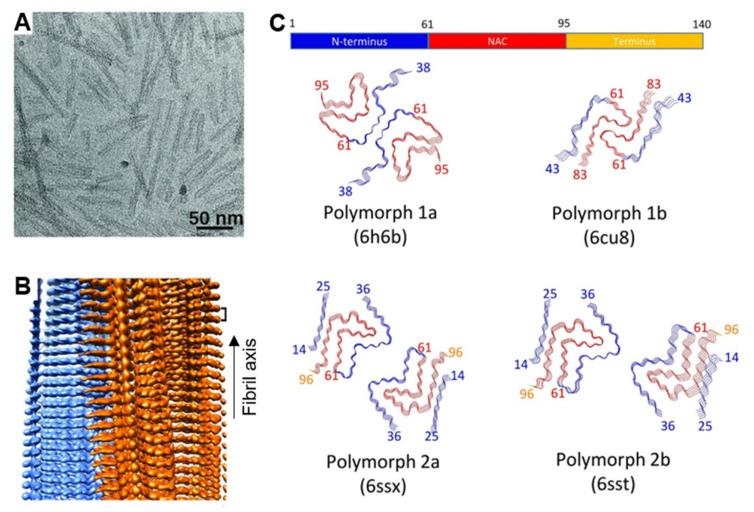
Structural features of αS fibrils. (**A**) Representative atomic force microscopy (AFM) and (**B**) 3D cryo-EM reconstruction image of a sample of αS fibrils. Adapted from Figure 1B,C, Guerrero-Ferreira, R. et al. 2018, eLife, published under the Creative Commons Attribution 4.0 International Public License (CC BY 4.0; https://creativecommons.org/licenses/by/4.0/) [123]. (**C**) Summary of the structural differences of αS fibril polymorphs resolved by cryo-EM. Reproduced from Figure 4, Guerrero-Ferreira, R. et al. 2019, eLife, published under the Creative Commons Attribution 4.0 International Public License (CC BY 4.0; https://creativecommons.org/licenses/by/4.0/) [119,120].

In the last few years, and with the great advances of solid-state nuclear magnetic resonance (ssNMR) and cryo-electron microscopy (cryo-EM) techniques, a number of structures of different αS fibril polymorphs generated in vitro with recombinant protein have been resolved at atomic resolution, and significant structural differences between the different fibril polymorphs have been observed. In some structures the protofilament fold is similar, typically forming a Greek key structure [121,122], with the differences arising in the packing of the protofilaments (the type of inter-protofilament interactions and mutual disposition between protofilaments) (see Figure 3). Most of the reported fibril structures contain the same central region of the protein as the amyloid core, including residues 35–100 [119,121,122,123]. The number and location of β-strands within this region, however, varies between fibril polymorphs generated under different solution conditions, with the ionic strength, the presence of polyanions or the pH of the solution as main factors for fibril structure variability. These observations, therefore, already highlight the relevance of electrostatic interactions in the intramolecular and intermolecular forces that define the overall structure in the different amyloid polymorphs [119,124,125]. Two regions within the amyloid core have been suggested to be important for stabilizing the amyloid structure [123], concretely 71–82 region, which was also found to be essential for the initiation of the aggregation process [126], and 45–57 region, where most of the pathological point mutations are located.

The large scale structural differences between amyloid polymorphs induced by small changes in buffer conditions [119] highlights the large variability of structural rearrangements and interaction networks able to stabilize an amyloid fold of the same protein. These different polymorphs have very similar overall free energies, which results in a flat amyloid misfolding landscape with many local minima. This landscape contrasts with the funnel-shaped landscape found for the folding of most proteins, where the folded, native structure represents the lowest global energy minimum [127,128]. Indeed, the type of landscape of the amyloid conformation is the consequence of the type of interactions that stabilize this particular conformation, which are dominated by hydrogen bonds between the main-chain atoms of the polypeptide chain [129]. The contributions of the interactions between side chain groups to the overall energy of the amyloid structure are typically less relevant, although they seem to be crucial for the definition of the type of amyloid polymorph preferred during the nucleation step, which is ultimately defined by the solution conditions.

In this scenario, it would seem very plausible that alternative amyloid polymorphs could be formed in different cellular types and perhaps in different microenvironments of the same cell. If amyloid aggregation would be a favorable process, the consequences would be that multiple amyloid polymorphs could co-exist even in the interior of the same cell. The large energetic barrier for primary nucleation makes typically αS aggregation a kinetically unfavorable process (under highly hydration conditions and in the absence of pre-formed fibrils). Once triggered, the faster rates of elongation and secondary processes, as compared to the rate of primary nucleation [118], would result in the prevalence of the polymorphs that multiply quicker, i.e., the system is typically governed by a kinetic rather than a thermodynamic partitioning. If fragmentation and elongation are the preferred secondary processes, then it could be possible that only one major type of polymorph dominates the amyloid population, which would be defined by the initial type of interactions that are established during primary nucleation. If, under other conditions, secondary nucleation is more favorable than elongation, with rates comparable with that of primary nucleation, then various types of polymorphs could co-exist, since the structure of the aggregates generated by secondary nucleation does not typically reflect the structure of their fibrillar parents [130]. The structure of the aggregates generated by secondary nucleation, in analogy to what is found for those formed under primary nucleation, depends mainly on the solution conditions [130]. These are important points that are not always taken into consideration when analyzing for example the structure of in vivo-generated amyloid aggregates by NMR or cryo-EM after amplifying the number of aggregates by in vitro seeding reactions. In such seeding reactions, secondary nucleation processes could be governing which would favor the formation of amyloid aggregates with structures significantly different from those of the parent fibrils. The predominance of one type of amyloid polymorph over others depends primarily, therefore, on the properties of the protein microenvironment, as those dictate the structure of the pre-nucleus during primary and secondary nucleation, as well as the relative rates of primary and secondary nucleation, and elongation. 

The same molecular event of αS amyloid aggregation is associated with not only PD but also other neurodegenerative disorders. In the particular case of MSA, the αS inclusions are localized in different cellular types with respect to the other synucleinopathies, which suggests that, at least for this disease, αS aggregation is likely triggered by an alternative mechanism to that associated with PD or DLB. In agreement with this idea, pathological αS in glial cytoplasmatic inclusions or in LBs has been reported to be conformationally and biologically distinct [17,35]. The intracellular environment is, therefore, determinant for the accumulation of particular types of amyloid polymorphs. These results are in line with earlier studies that suggested the existence of disease-specific αS amyloid polymorphs. In a seminal study, injection of amyloid-like fibrillar structures derived from MSA patient´s brain extracts into experimental animals induced neurodegeneration accompanied by αS deposition in all cases. However, none of the animals inoculated with αS fibrillar species obtained from PD brains developed neurological deficits [17,35]. Parallel studies were performed with in vitro-generated amyloid polymorphs and showed the variety of seeding capacities and neurotoxic properties of the different αS aggregated species analyzed [18,131]. Recently, the structure of αS inclusions extracted from the putamen of deceased MSA patients was compared to those extracted from the cortex and amygdala of deceased DLB patients by means of cryo-EM. All the MSA patients analyzed showed very similar filament structures, which were very different from the filaments extracted from DLB cases, and in both cases remarkably different from those that have been formed in vitro up to date with recombinant proteins [132]. These findings highlight the urgent need to find αS aggregation conditions in vitro that truly recapitulate the aggregation pathways triggered in vivo. Despite the similar amyloid folds, two types of αS filament polymorphs in MSA brains were distinguished, being the so-called Type-II structure preferred in patients with a longer life span [132]. An interesting feature observed in MSA patients’ brain derived fibrils was the presence of an additional molecule, of yet unknown nature, that connects the two protofilaments in both Type I and Type II filaments [132]. This molecule establishes electrostatic interactions with positively charged residues in αS, specifically the side chains of residues K43, K45 and H50. These interactions might be strongly altered by the G51D and A53E disease-associated αS mutations, which could lead to altered fibrillar structures and stabilities, which, in turn, could be related to the characteristics of these hereditary forms: early disease onset, shorter disease duration (compared to sporadic PD) and severe cognitive and psychiatric disturbances. Overall, these findings are in line with the idea that different conformations of αS contribute to the type of neurodegenerative disease as well as disease prognosis. 

Along the most recent improvements regarding identifying and studying the variety of amyloid polymorphs in synucleinopathies, protein misfolding cyclic amplification (PMCA) [133] together with real-time quaking-induced conversion (RT-QuIC) [134] are providing highly valuable insights into the characteristics of patient-derived αS aggregated species as well as helping to bring about new diagnostic tools in the field of neurodegeneration [135]. Both techniques allow replicating and amplifying the structure of abnormal αS by repeatedly fragmenting the parent aggregates and using them as templates for seeding reactions with recombinant monomeric αS in the presence of the thioflavin T probe. By comparing the seeding kinetics, important information about the seeding efficiency of patient-derived or in vitro-generated amyloid polymorphs can be extracted. Both assays have been used with fibrillar material extracted from brain or gastrointestinal tissue of PD, MSA and DLB patients and the aggregates generated were demonstrated to faithfully recapitulate the biochemical, structural and neurotoxic properties of the parent, in vivo-generated aggregates [136,137,138,139,140,141,142]. These assays can distinguish between cerebrospinal fluid samples from patients diagnosed with PD or MSA [143], and are being further developed with the aim of using them as diagnostic and prognostic tools for the distinct types of synucleinopathies. 

### 4.2. Oligomeric Polymorphs

The process of amyloid fibril formation requires the transition of the protein by a series of multiple oligomeric intermediates that differ in terms of size (from dimers to large assemblies containing typically less than a hundred of protein units) and structure (from essentially disordered structures similar to those of their monomer precursors to β-sheet rich structures close to that displayed in the fully formed fibrillar state). The distinction between oligomers and short fibrils is not always trivial and size alone might not be a good parameter for this. A distinctive characteristic of fibrils is that they elongate from their ends by association of monomers from the solution and that the elongated structures have the same structure and β-sheet content as their aggregate parents. In contrast, the elongation of oligomers would result in more elongated structures with a significantly higher β-sheet content. A critical parameter, therefore, for the definition of oligomers is the degree of β-sheet content as compared to that of the fibrillar structures, which should be used in addition to the size of the aggregates.

Given the multiplicity of types of oligomers in route to fibril formation and their inherent transient intermediate nature in the process, their study and characterization have been proved to be extremely challenging. Single-molecule techniques, however, have provided valuable information of such species. Two structural groups of αS oligomers formed during the lag-phase of fibril formation under typical in vitro reactions (i.e., under conditions of heterogeneous nucleation at the air/water interface) were identified by single-molecule Förster resonance energy transfer [10]. The initially formed oligomers present a diffuse, proteases sensitive and non-toxic structure (referred to as type-A oligomers). These oligomers slowly convert into more compact and stable, protease resistant, toxic oligomers (referred to as type-B oligomers), with partially-formed β-sheet structure. These oligomers elongate later through monomer addition to form protofilaments and eventually mature fibrils (see Figure 1) [10,144]. In order to study these different structural oligomeric forms further, we and others have developed strategies to prepare samples of analogous stable oligomeric forms that result in more than 90% of the sample enriched in each particular type of oligomer. These oligomeric samples have been characterized in detail and have been reported to be stable for days. 

The strategy used to prepare samples enriched in a type of oligomers analogous to type-B oligomers consists in inducing the aggregation of the protein through a particular amyloid pathway at dehydrating conditions (i.e., during lyophilization), under which homogeneous nucleation is favored [92]. This pathway results in the accumulation of kinetically trapped, antiparallel β-sheet, toxic oligomers whose rates of disaggregation into monomers and elongation into fibrils are extremely slow. These oligomers were referred to as type-B* oligomers to remark that they are analogous to type-B oligomers but not exactly the same, as type-B oligomers are generated during the heterogeneous primary nucleation of parallel β-sheet amyloid fibrils [103]. Despite the differences in the β-sheet arrangement of the amyloid structure between type-B and type-B*oligomers, they are similar in a number of features, reflecting their analogous nature. Both types of oligomers have similar sizes, an intermediate secondary structure between the monomeric and the fully-formed mature fibrils, similar affinities for lipid membranes and similar toxic mechanisms, with apparently identical cellular dysfunction effects [10,26,27,103,145]. One remarkable difference is their ability to elongate: while type-B oligomers quickly elongate into fully-formed fibrils, type-B* oligomers have their elongation abilities disrupted, likely as a result of the antiparallel intermolecular β-sheet structure. Other amyloid-like aggregates with an antiparallel β-sheet structure show a preference for small aggregates rather than the typical parallel β-sheet amyloid aggregates with long fibrillar morphologies [92]. Therefore, caution is needed when extrapolating to the type-B oligomers the biological effects identified for type-B*, or the analogous situation in other amyloidogenic proteins or peptides. Both types of oligomers seem to behave similarly in terms of induction of toxicity, but in terms of seeding properties, and therefore spreading capabilities, they behave very differently.

In the case of the oligomers analogous to type-A, formed during the heterogeneous primary nucleation of the protein, the stabilization strategy consisted in using a molecule (epigallocatechin gallate, EGCG) that binds to this structural type of oligomer and prevents its structural conversion and progression into type-B oligomers and eventually amyloid fibrils [145]. We referred to these oligomers trapped by the action of an inhibitor molecule that remains bound to the aggregates as type-A* oligomers to reflect the fact that they consist on a complex between type-A oligomers and several molecules of EGCG. Additionally, EGCG has been shown to modify chemically αS but without major effects on oligomer formation [146]. Both type-A and type-A* oligomers have similar sizes, have a disordered structure and are benign to the cells [10,145], but the presence of several molecules of EGCG in the surface of the oligomer might affect some properties with respect to the unbound oligomer form.

**Figure 4:**
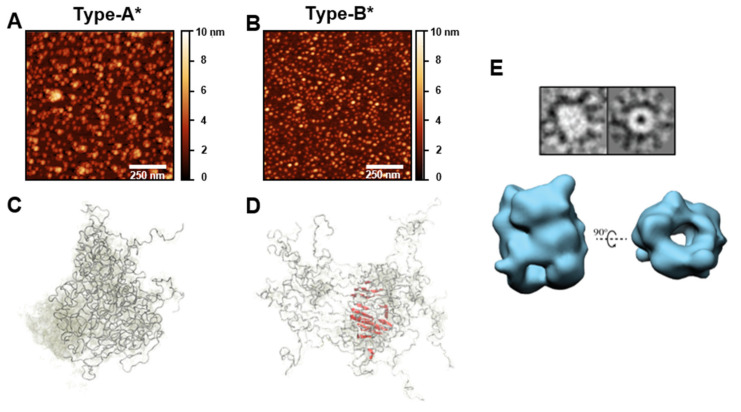
Structural features of two structurally different types of αS oligomers. Some of the most detailed structural information on αS oligomers has been obtained by the use of enriched samples of structurally homogeneous oligomeric species that have been trapped by the addition of molecules that prevent the conversion of oligomers to fibrils (for example the molecule EGCG, that results in the accumulation of type-A* oligomers) or the induction of alternative amyloid pathways by the modulation of the solution conditions that result in the kinetic stabilization of oligomeric species (i.e., induction of homogeneous nucleation under limited hydration conditions such as lyophilization, which results in the formation of type-B* oligomers). (**A**,**B**) Typical morphology of type-A* oligomers (**A**) and type-B* oligomers (**B**) probed by AFM. (**C**,**D**) Structural models of type-A* oligomers (**C**) and type-B* oligomers (**D**) according to the information obtained from solution and ssNMR data (from Fusco et al., *Science* 2017, reprinted with permission from AAAS) [145]. (**E**) Morphology and 3D-reconstruction models of type-B* oligomers according to cryo-EM image analysis (reprinted from Chen et al., PNAS 2015) [103].

Both type-A* and type-B* oligomers have similar size distributions, with the majority of the species showing sizes of ca. 15–40-mers (for the type-B* oligomers an average of ca. 30 protein molecules was estimated) [103,145]. The vast majority of the species in both samples present a rather homogeneous structure: essentially disordered in the case of type-A* oligomers, and with a β-sheet core composed of 70–88 residues in the case of type-B* oligomers. Indeed, the structural homogeneity of both oligomeric samples have allowed their structural characterization by ssNMR [145] and the 3D cryo-EM structural reconstruction in the case of type-B* oligomers (at 18-Å resolution) [103] (see Figure 4). The structural reconstruction by cryo-EM showed that the oligomers presented a cylindrical morphology, similar to that previously reported for toxic prefibrillar oligomers [147], with a diameter of ca. 10 nm, that coincide with the diameter found for αS mature fibrils. However, in contrast to the αS fibrils whose structure has been determined up to date, these oligomers show a hollow core, suggesting that the interactions between β-sheets are predominantly hydrophilic and mediated by water molecules. This structural arrangement results in a significant fraction of hydrophobic residues exposed to the solvent at the surface of the oligomer structure [103], which explains the particular ability of this type of oligomer to interact with the interior of the lipid bilayers of cellular membranes and disrupt membrane integrity, leading to the toxic consequences for the cell [145]. Similar water-mediated protofilament interactions with the concomitant formation of fibrils with a hollow-core have been reported for other amyloidogenic polypeptides related to disease [5,8,148]. In the case of αS fibrils, all the structures obtained by cryo-EM analysis and composed of multiple protofilaments (typically two) show a hydrophobic interface between protofilaments that is stabilized by a few ionic salt bridges. This protofilament arrangement results in most of the hydrophobic residues of the protein buried in the interior of the mature fibrils and a fibrillar surface mostly hydrophilic. In contrast, a highly hydrophobic fibrillar surface is inferred, indeed similar to that of the type-B* oligomers, when the nature of the solvent exposed surface of analogous fibrils was analyzed and compared with monomeric and Type-A* oligomers using the 8-anilino-1-naphthalene sulfonate (ANS) probe [103,149]. This apparent contradiction needs to be resolved, as the solvent-exposed hydrophobic surface seems to be highly correlated with toxicity in amyloid aggregates [150].

Other αS oligomeric structures that have been reported to be formed during fibril formation include annular oligomers, as visualized by transmission electron microscopy (TEM) and AFM [151,152], but not further structural determination was reported for such oligomers and their relationships with the fibrillar structures and other types of oligomers remain unknown. Oligomeric forms generated during fibril formation have been isolated, in some cases purified by gel filtration, and remain stable under a variety of conditions, which allowed certain structural and mechanistic characterization [101,153,154,155,156], with some features, such as size (average of 29–31 monomers per oligomer), antiparallel β-sheet structure [101] and amyloid core regions [153], reminiscent to those of the type-B* oligomers. 

A large number of αS oligomeric species have been described as a result of their stabilization by particular types of molecules able to either bind to or covalently modify αS, such as selegiline [153], baicalein [157,158] or dopamine and its analogs [159,160]. Although significant differences in morphology, size and secondary structure for the different αS oligomeric species have been shown, a general trend is observed. Most of the reported stabilized oligomers were either mainly disordered, which were generally reported to be nontoxic to neuronal cells, or presented certain, but limited, β-sheet structure, which showed significant level of toxicity in cells [120], suggesting that these two main types of oligomers could be general to the various αS aggregation pathways that have been explored up to date.

## 5. Possible Roles of α-Synuclein Species in Cell Toxicity

Aberrant folding and aggregation of certain amyloidogenic proteins has been suggested to be the initial trigger of amyloid diseases, later followed by a cascade of events such as calcium and metal ion dyshomeostasis, oxidative stress and proteostasis impairment. This initial trigger has been related with the ability of certain aggregated species to bind and disrupt cellular membranes. Amyloid aggregates from different proteins and peptides, including some not related *a priori* with human diseases, have been shown to induce cellular toxicity by similar mechanisms of membrane perturbation [161,162]. These experimental observations suggested that this mechanism of toxicity is indeed a common feature for this type of aggregates that share the same toxic structural determinants [1,13]. While the intrinsic toxic properties of the aggregates are independent of cellular proteostasis, their accumulation and life span strongly depends on the cellular proteostasis activity [163].

The nature of the most toxic aggregated species formed during amyloid aggregation, in particular for αS, remains a subject of intense debate in the field. Both oligomers and fibrils have been shown to be toxic in different contexts and comparative studies have reported contradictory results, although the nature of the aggregates, particularly for the oligomeric species, is generally not defined with the precision required to derive strong conclusions. There is multiple experimental evidence for an important role of particular types of oligomers in αS amyloid toxicity. Structural-toxicity studies on in vitro-generated αS oligomers have generally shown that disordered oligomers are benign to cells, while oligomers with partially formed β-sheet cores and highly hydrophobic surfaces are the most inherently toxic αS species upon exposure to cells [120]. While these studies were performed with in vitro-generated αS aggregates, similar cellular effects were observed in a human iPS-derived αS triplication neuronal model with endogenous αS aggregation, suggesting that analogous types of αS aggregated species are also formed in vivo [26,27]. The ability of the rudimentary cross-β oligomers to induce toxicity has been proposed to arise, at least in part, from their ability to bind lipid membranes and insert their flexible, hydrophobic β-sheet core into the interior of the lipid bilayer [145]. The combination of these structural features is absent in the monomeric and early disordered oligomeric forms [145,164], as well as in the highly rigid (in some cases with rigidities close to steel [165]) fibrillar structure. The insertion of this type of oligomers into the lipid membranes causes, then, a cascade of toxic effects in the cells such as calcium and metal ion imbalance, oxidative stress, mitochondrial and lysosomal dysfunction, and eventually cell death [26,27,166,167,168,169]. In addition, the exposure of hydrophobic residues and hydrogen-bond unsatisfied amino acid groups on the surface of these inherently intermediate, partially folded species, likely result in the aberrant and promiscuous interaction of these species with other proteins and cellular components that could disrupt their correct biological functions. This situation would explain some of the reported cellular effects of αS oligomers such as synaptosomal and mitochondria dysfunction [170,171] and the impairment of the ubiquitin-proteasomal systems [172,173]. 

Although αS fibrillar aggregates are able to bind to cellular membranes, they appear to remain bound to the membrane without inserting their β-sheet core into the lipid membrane, in contrast to the toxic β-sheet oligomers [145] and, consequently, their effects on membrane perturbation are milder in terms of mass concentration, unless a massive overload of fibrils are bound to the same cell at the same time. However, similar qualitative effects, such as intracellular ion homeostasis perturbation [174] and cellular proteostasis [173], mitochondria [175] and lysosome dysfunction [176], have been observed for both fibrillar and β-sheet oligomers, suggesting possible common mechanisms of toxicity between oligomeric and fibrillar species. Recent evidence shows that distinct fibrillar αS polymorphs cluster at the plasma membrane of neurons to a clearly different extent, and also exhibit different seeding abilities. These results were interpreted in terms of variations in the organization of membrane receptors induced by distinctive types of αS fibrillar polymorphs [177]. In some reports, αS fibrils have been shown to induce an inflammatory response [178,179], although further studies are required to elucidate the role of αS aggregates on the induction of a chronic inflammation, proposed to be an important factor in triggering neurodegeneration.

## 6. Possible Roles of α-Synuclein Species in Cell-to-Cell Propagation

Misfolded forms of αS, and other proteins associated with neurodegenerative disorders, have been shown to self-propagate and spread between interconnected regions of the central nervous system acting as infectious agents propagating neurodegeneration, in a similar manner as it has been described for prions [179]. The spatiotemporal progression of αS pathology and the progressive nature of PD and other synucleinopathies [179] suggests the presence of specific anatomic αS spreading pathways.

Cell-to-cell transmission of αS aggregates has been experimentally observed [179] and more directly evidenced in grafted fetal mesencephalic neurons in PD patients after a host-to-graft αS misfolded transmission [180,181]. Indeed, the αS pathology transmission model has been used to develop cell and animal models of αS pathology as the exposure of neurons to extracellular pre-formed αS fibrils induces intracellular endogenous inclusions resembling those found in disease brains, the loss of dopaminergic neurons in the SNc and the associated motor-behavior defects, recapitulating the core features of PD [75].

We recently published a study that compared the ability of short (50 nm) fibrils and seeding-incompetent oligomers (type-B* oligomers) to cause PD-related phenotypes in mice. Striatal injections of short fibrils or elongation-deficient oligomers caused loss of dopamine neurons in the SNc [75]. The short fibrils caused also recruitment of endogenous αS into pS129-positive inclusions that resembled LB pathology, loss of dopamine terminals in the striatum, and induction of motor behavior phenotypes. However, the seeding-incompetent oligomers caused some loss of dopamine neurons in the SNc, reflecting their toxicity, but they did not produce αSyn inclusions or behavioral phenotypes. Short amyloid fibrils, and likely elongation-efficient oligomers (type-B oligomers) that can recruit and corrupt endogenous αS, are responsible for the majority of these phenotypes. Long fibrils, however, were unable to internalize efficiently and therefore spread from cell to cell. These results highlight the role of the size of the amyloid aggregates in the induction of intraneuronal αS inclusions, and suggest a potential toxic effect of those processes that could occur in the cell and result in the accumulation of small amyloid-like aggregates, such as fibril fragmentation, fibril disaggregation or uncompleted aggregation. One example is the action of the Hsp70 chaperone disaggregation system that has been reported to be able to either reduce αS aggregation and spreading [182] or enhance αS amyloid aggregation by releasing spread-competent αS species [182]. The in vitro Hsp70-mediated disaggregation of αS fibrils results primarily in the release of monomeric protein units from the fibrillar ends [182]. Under optimal conditions (optimal chaperone-to-co-chaperone ratios and ATP availability), the chaperone system is highly efficient and, therefore, the lifetime of possible toxic intermediate species generated during the disaggregation process (either very short fibrils or oligomers) would be very short and the overall Hsp70 system disaggregation activity would be beneficial to eliminate αS toxicity [182]. However, if the disaggregation process is not completed because some of the components of the chaperone complex are not optimal or the ATP availability is very limited at some point of the reaction in the cell, this could result in the transient generation of toxic short fibrils and oligomers which could then diffuse and cause aberrant interactions with cellular components and spread efficiently from cell to cell, as proposed in [183]. 

Disease-related familial mutations could alter the population distribution of amyloid polymorphs, which could be related with their potential contribution to the pathogenesis [122,125]. Initial cryo-EM structures of WT αS fibrils indicated that at least four of the five mutation sites found in familiar PD (E46K, A53T/E, G51D and H50Q) are preferentially located at the protofilament interfaces, rather than at the kernel of the amyloid structures. The presence of such mutations were suggested to affect severely the protofilament arrangement and then the stability of mature fibrils, which was correlated with the potential increase in the fraction of toxic oligomeric species within the cells [125]. The E46K pathological mutation, however, was later reported to adopt a significantly different amyloid fold as compared to the amyloid structure adopted by the WT protein at similar conditions [124,125]. The acquisition of this alternative fibril kernel conformation by the E46K mutation results in a significantly higher propensity to seed the formation of new fibrils as compared to the WT fibrils [124,125], which could influence the speed of αS pathology spreading. It is interesting to note that the E46K mutation seems to be the only hereditary αS mutation that manifests in a clinical picture closer to DLB; the other mutations being more associated to PD.

## 7. Post-Translational Modifications

αS has a number of PTMs, with phosphorylation, ubiquitination and nitration highly related to pathology [184]. Phosphorylation has been identified on various serine and tyrosine residues, mostly located at the C-terminal region of the protein. Despite being the most PTM studied in αS, its role in physiology and disease remain controversial [185]. Phosphorylation on serine 87 (pS87-αS), in the NAC region, appears to be increased in PD brains but, paradoxically, reduces the membrane-interaction ability and blocks αS fibril formation in vitro [186]. Other studies using a rat model, however, point toward a neuroprotective effect of pS87-αS [187]. The phosphorylation of some residues such as tyrosine 125 (p125-αS) has been reported to have a beneficial role regarding neurotoxicity [188], while the same modification at serine 129 (pS129-αS) is indicative of pathology. It has been found that more than 90% of the αS in the LBs of PD patients´ brains is phosphorylated at serine 129 [184], while only ca. 4% in healthy brains [184]. This particular PTM has been shown to increase the rate of αS amyloid aggregation [184], as well as the toxicity associated with this process [189]. Although other studies have suggested a protective role based on its influence in reducing αS membrane binding [190] and increasing the conformational flexibility of the monomeric protein (which would prevent fibril formation) [191]. In the same line, constitutively expressed pS129-αS did not induce neurotoxicity in an overexpression rat model, while non-phosphorylated αS did [192].

Another important type of PTM in αS is the ubiquitination of lysine residues located at the N-terminal region of the protein. This region typically falls outside the amyloid core, although its ubiquitination has been observed to inhibit amyloid formation [193]. Significant steric repulsions for amyloid formation and structural differences in the fibrils would be expected upon ubiquitination in this region of the protein given the size of ubiquitin with respect to that of the αS. Indeed, this PTM can occur downstream fibril formation, given that most of the lysine residues of the N-terminal region are exposed to the solvent and readily accessible for ubiquitination according to the currently available cryo-EM structures [194]. 

Nitration of certain tyrosine residues are also commonly observed in pathologically relevant αS inclusions and in some cases, such as nitration of tyrosine 39, have been reported to stabilize soluble oligomeric species via dityrosine crosslinking [195]. 

The only available atomic structure of a post-translationally modified αS fibril carries the N-terminally acetylated modification, and no differences with the unmodified protein fibrils were observed [122]. This PTM is a mild modification located at a very distant region of the amyloid core in the amyloid fibrils. It would be relevant to determine the influence of other PTMs, particularly those related to pathology, on the stability and structure of the αS oligomers and fibrils and their effect on cells, as well as to define whether these modifications occur primarily before or after αS oligomerization.

## 8. Summary

It is gradually being accepted in the field that certain types of soluble oligomeric species of αS are more inherently toxic than larger fibrillar aggregates. These oligomers can be generated *de novo* from cytosolic αS or αS associated with membranes. However, recent experimental evidence shows that fibrils and large intracellular inclusions can also be a source of soluble oligomers by fibril disaggregation [10] and secondary nucleation processes [32], which could explain, at least in part, the similarities in the toxic effects observed between oligomeric and fibrillar species. Mutations in αS that destabilize the dimeric interface of mature fibrils, such as the pathological mutations H50Q, G51D and A53T/V/E, may lead to a larger population of soluble toxic oligomers. However, these mutations are extremely rare, so understanding the cellular mechanisms which favor β-sheet oligomer formation need to be further elucidated. These can include: slight increases in αS levels, defects in lysosome activity, oxidation and PTMs that can occur particularly in dopamine neurons, induction of the formation of intracellular αS droplets by LLPS, aberrant activity of chaperone systems or expression of other mutant genes that can impact any of the above processes. Templated recruitment of endogenous αS also occurs and, for this, particularly short fibrils appear to be the most potent αS species [75]. Multiple types of aggregates are, therefore, populated during the process of self-assembly and might be involved in different stages of the development of pathology, some species being directly involved in the induction of neurotoxicity and others in the propagation of pathology [77].

A combination of three parameters appear to be key for the potential toxicity of αS aggregates, and likely aggregates from other amyloidogenic proteins: size, a highly exposed hydrophobic surface, and structural flexibility. Recent experimental evidence of remarkably different seeding capabilities of structurally alternative amyloid polymorphs suggests that other structural properties play also a role in αS aggregate toxicity. Further studies on structure-toxicity relationships on different αS aggregate polymorphs will help to define the molecular basis of toxicity and spreading in αS aggregates. It is important to identify also the structural determinants in the aggregates that dictates the preference for the recruitment of monomeric molecules either at the ends of the fibrillar structure or at its longitudinal surface and, thus, for the type of secondary process that governs the system, as this is an important source of polymorph variability. Further experimentation is required to (i) elucidate the detail structure of soluble toxic oligomers and the polymorphism associated with this type of αS species, (ii) how solution conditions, pathological mutations and PTMs impact on their structure and stability and (iii) the type of oligomer and fibril polymorphs that form in vivo in a disease-related context and that could be associated with the distinct types of synucleinopathies. The type of αS fibril polymorphs isolated from patient-derived brain samples are remarkably different from the fibril polymorphs that up to date have been generated in vitro and whose structures have been characterized [132,196]. It would be relevant to characterize the structural types of αS oligomers and fibrils that are formed in alternative conditions, and therefore through alternative amyloid pathways, to those explored in vitro. In particular, those generated through liquid-to-solid transitions inside αS droplets generated by LLPS, which have been recently suggested to precede αS amyloid formation in vivo, and that are likely to occur through very different amyloid mechanisms to those of the typical processes studied in vitro under diluted and highly hydrated conditions [92]. Gaining a better understanding of the type of amyloid pathways and primary and secondary processes occurring in vivo, as well as the nature and structure of the types of polymorphs of oligomers and fibrils formed, will help not only to identify the molecular basis of αS aggregation in disease, but also to design future treatments and develop new diagnostic tools for the early diagnosis of distinct types of synucleinopathies.
